# Pharmacokinetic Alterations in Patients with Chronic Heart Failure: A Systematic Review

**DOI:** 10.3390/ijms26199495

**Published:** 2025-09-28

**Authors:** Olga Butranova, Sergey Zyryanov, Yury Kustov

**Affiliations:** 1 Department of General and Clinical Pharmacology, Peoples’ Friendship University of Russia Named After Patrice Lumumba (RUDN), 6 Miklukho-Maklaya St., 117198 Moscow, Russia; zyryanov_sk@pfur.ru (S.Z.); 1142240146@pfur.ru (Y.K.); 2 Moscow City Health Department, City Clinical Hospital No. 24, State Budgetary Institution of Healthcare of the City of Moscow, Pistzovaya Str. 10, 127015 Moscow, Russia

**Keywords:** chronic heart failure, pharmacokinetic parameters, cardiovascular drugs, ACE inhibitors, lisinopril, loop diuretics, furosemide, PDE inhibitors, milrinone, digoxin

## Abstract

(1) Chronic heart failure (CHF) is a typical component of the polymorbid profile of an elderly patient. The aim of this systematic review was to search for data from pharmacokinetic (PK) studies of any drugs in patients with CHF to systematize information on changes in PK parameters depending on the physicochemical properties (PCPs) of the drug and route of its administration. (2) A systematic review of PK studies in patients with CHF was performed using Elibrary.ru, United States National Library of Medicine (PubMed), China National Knowledge Infrastructure (CNKI), and Directory of Open Access Journals (DOAJ). The final number of included articles was 106. A descriptive and correlation analysis of PK data and PCPs of drugs included in the study was carried out. Inclusion criteria: PK study, available PK parameters, demographic data, and diagnosed CHF. Risk of bias was assessed using ROBINS-I. (3) Evaluation of correlations between PCPs of drugs and their PK revealed a link between (i) plasma protein binding (PPB) and volume of distribution for lipophilic drugs; (ii) PCPs, half-life, and clearance for drugs with high PPB; and (iii) PPB and clearance for hydrophilic and amphiphilic drugs. (4) Hypoalbuminemia associated with CHF may lead to an increased volume of distribution of lipophilic drugs; lipophilic drugs used in CHF patients may be associated with prolongation of the half-life period and reduction in clearance; highly protein-bound drugs may manifest with reduced clearance. PK characteristics identified in this review should guide modifications to dosing regimens in CHF patients receiving medications from different groups.

## 1. Introduction

CHF is among the most common chronic diseases worldwide. According to data from S. Jimenez et al. (2020), CHF prevalence in the European population is 2–4% [1]. The highest prevalence is typically detected in the elderly, from 7.7 to 9% [2]. According to the NHANES study (2017–2020), there is a trend toward increasing CHF prevalence in the American population aged over 20 years, reaching up to 8.7 million by 2030 [3]. The combination of pathological changes specific to CHF patients can affect drug efficacy and safety due to alterations of all PK processes: absorption, distribution, metabolism, and excretion.

In a study by Osadchiy V.A. et al. (2015), it was found that patients with CHF (NYHA functional classes I, II, and III) exhibited reduced gastric hydrochloric acid secretion due to impaired blood flow [4]. Decreased acid production by gastric parietal cells with subsequent alkalization of the gastric environment may lead to reduced solubility and absorption of drugs. A representative example is itraconazole, absorption of which decreases by 65% under achlorhydric conditions [5]. Intestinal venous congestion causes alterations in the gut microbiome [6], which can subsequently affect the absorption and metabolism of some drugs [7].

Fluid redistribution typical for CHF patients results in increased extracellular fluid compartment [8]. Consequently, the number of fluid compartments available for drug distribution expands, potentially altering drugs volume of distribution. CHF-induced hemodynamic changes may lead to congestive hepatopathy, liver fibrosis, and non-alcoholic fatty liver disease, impairing hepatic protein synthesis and consequently reducing albumin levels [9,10,11,12]. Hypoalbuminemia decreases protein-bound drug fractions, increasing pharmacologically active (unbound) drug concentrations. Elevated unbound fraction may mediate increased risks of toxicity and adverse drug reactions (ADRs). Another reason for increased risks of toxic drug effects in CHF patients may be related to the known down-regulation of hepatic CYP enzymes specific to the given population. CHF mediates hepatocyte damage, hypoxemia, increased pro-inflammatory cytokine levels, and heightened production of heme oxygenase-1 [13].

CHF-associated renal parenchymal hypoperfusion decreases drug clearance and prolongs elimination half-life, potentially leading to drug accumulation and subsequent ADRs [14].

The sum of PK changes unique to CHF patients may lead to significant variability in drug plasma levels, which can result in diminished efficacy of pharmacotherapy [15]. Existing PK studies in CHF patients are primarily focused on cardiovascular drugs, with limited data on other pharmacologic classes. The aim of this systematic review was to identify PK studies of various drugs in patients with CHF and to systematize information on PK parameter alterations based on drugs’ PCPs and administration routes.

## 2. Materials and Methods

Object of the study—Elibrary.ru, United States National Library of Medicine (PubMed), China National Knowledge Infrastructure (CNKI), and Directory of Open Access Journals (DOAJ) databases.

Search period: lower date limit—not applied, upper date limit—1 March 2025.

Search language: Russian and English.

Keywords for Elibrary.ru database search: “chronic heart failure”, “congestive heart failure”, “pharmacokinetics”, “clearance”, “half-life”, “volume of distribution”, “pharmacokinetic parameters”.

Keywords for PubMed, CNKI, DOAJ database search: (congestive heart failure OR chronic heart failure OR CHF) AND pharmacokinetics AND (clearance OR half-life OR volume of distribution) AND pharmacokinetic trial AND pharmacokinetic parameters.

**Inclusion criteria:**

- Full article text access;
- Study type: PK study;
- Study population: CHF patients;
- Detailed reporting of PK parameters for the studied drug;
- Age ≥18 years;
- Language of the article: Russian or English.

**Exclusion criteria:**

- Studies lacking reported PK parameters of drugs;
- Publication types: narrative reviews, case–control studies, meta-analyses, and systematic reviews.

Our systematic review was conducted in accordance with the PRISMA (Preferred Reporting Items for Systematic Reviews and Meta-Analyses) guidelines [16].

**Figure 1:**
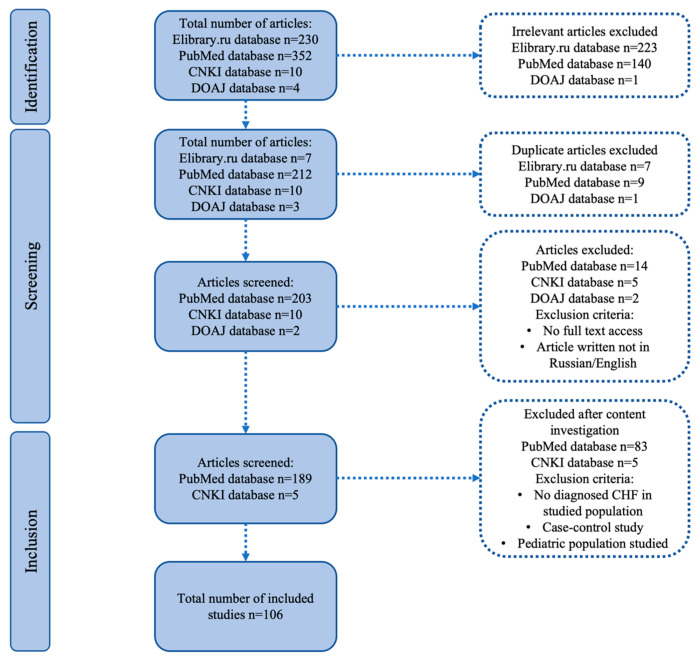
PRISMA flowchart.

Following the application of inclusion/exclusion criteria, 106 publications were selected for inclusion in this systematic review. The PRISMA flow chart is presented in Figure 1.

**Data identification:**

Three independent authors extracted the following data from eligible studies:

1. Demographic data: patients’ age, number of study participants, control group size (where applicable), NYHA functional class, and left ventricular ejection fraction (LVEF).
2. PK parameters: clearance, volume of distribution, half-life, and their alterations in CHF.

**Risk of bias assessment:**

Two authors evaluated the risk of bias using the Cochrane tool for assessing risk of bias (ROBINS-I).

**Statistical analysis:**

To assess correlations between physicochemical and pharmacokinetic drug properties, Spearman’s rank correlation coefficient was employed. A *p*-value of <0.05 was considered statistically significant.

## 3. Results

### 3.1. Study Identification

The initial number of publications was 596: 230 from Elibrary.ru, 352 from PubMed, 10 from CNKI, and 4 from DOAJ. After excluding invalid studies, duplicates, articles with no full-text access, and non-Russian/English publications, 189 articles from PubMed and 5 from CNKI remained. Further exclusion of studies without a confirmed CHF diagnosis in the study group, case–control studies, and those with pediatric populations resulted in 106 articles.

### 3.2. Quality Assessment

Two authors evaluated the risk of bias using the Cochrane tool for assessing risk of bias (ROBINS-I). The majority of the studies (n = 71; 67.0%) revealed a moderate risk of bias, 13 (12.3%) revealed a low risk, and 21 (20.8%) revealed a serious risk. Detailed results can be seen in Figure S1.

### 3.3. Study Characteristics

The search encompassed the entire period of database existence up to 1 March 2025.

The earliest identified publication date: 28 March 1974.

The most recent publication date: 25 March 2022.

The total number of included studies was 106, of which 13 (12.26%) contained data on more than one drug.

### 3.4. Drug Group Categorization

**Figure 2:**
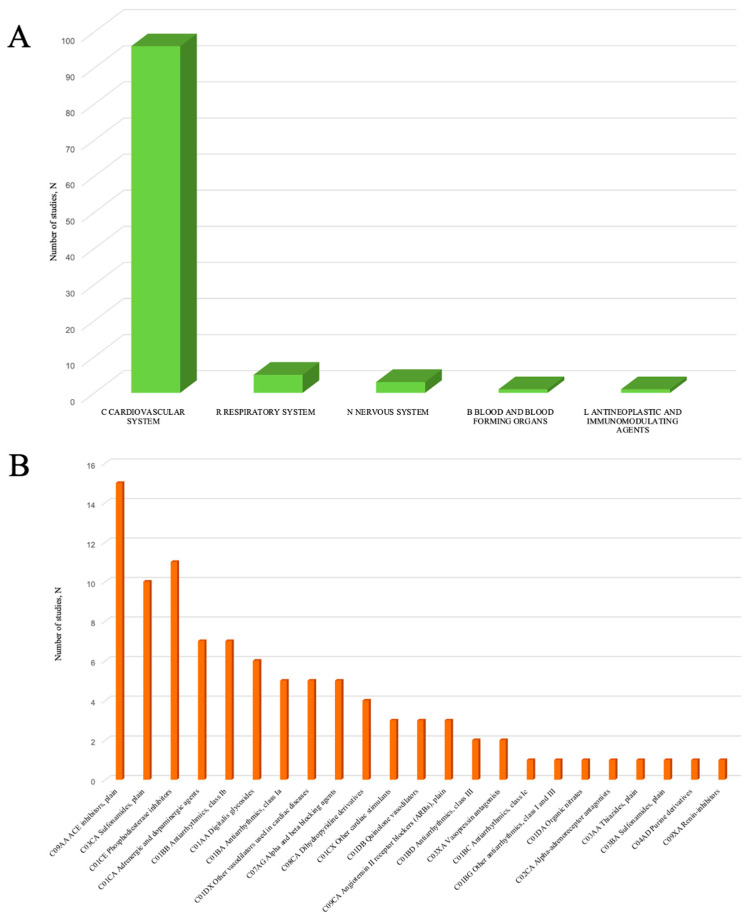
ATC groups revealed in PK studies in patients with CHF. (**A**)—structure of ATC Drug Groups (C—cardiovascular system, R—respiratory system, L—antineoplastic and immunomodulating agents, N—nervous system, and B—blood and blood-forming organs); (**B**)—structure of subgroups in ATC group C.

The first step in analysis was to classify all the drugs identified in PK studies into different groups using the Anatomical Therapeutic Chemical (ATC) classification system. Within ATC classification, substances are divided into groups according to the affected organ or system, therapeutic, pharmacological, and chemical properties (Table 1 and Figure 2). Detailed data on the structure of subgroups within ATC group C is presented in Figure 2B.

The total number of drugs identified in PK studies included in our systematic review was 71 (full description is given in Table S1). Evaluating the number of publications dedicated to each drug, we found that the largest number of publications was reported for furosemide (n = 9, 2 of which also contained data on bumetanide), digoxin (n = 6), theophylline (n = 5), and milrinone (n = 4).

The leading group was represented by cardiovascular drugs (Group C), accounting for up to 96 studies (90.6%). Within this group, the subgroups with the largest number of studies were angiotensin-converting enzyme (ACE) inhibitors (C09AA) (n = 15; 15.6%), loop diuretics (C03CA) (n = 10; 10.4%), phosphodiesterase (PDE) inhibitors (C01CE) (n = 11; 11.5%), adrenergic agents (C01CA) (n = 7; 7.3%), and class Ib antiarrhythmics (C01BB) (n = 7; 7.3%). The structure of drug subgroups in Group C is presented in Figure 2B and Table S2.

Among ACE inhibitors (C09AA), lisinopril (n = 3) and captopril (n = 2) were leaders based on the number of studies. Considering loop diuretics (C03CA), the largest number of studies was identified for furosemide (n = 9) and torasemide (n = 3). Among PDE inhibitors (C01CE), milrinone (n = 4) and enoximone (n = 3) were leaders. Studying adrenergic drugs, the largest number of studies was found for ibopamine (n = 3) and prenalterol (n = 2). The detailed structure of drugs in Group C is presented in Table S3.

Group R was presented only with theophylline (n = 5).

Group N drugs included acetaminophen (n = 1), midazolam (n = 1), and fluvoxamine (n = 1).

Group L contained only one study with etanercept.

### 3.5. PCPs Evaluation

The second phase of our study was focused on evaluating PCPs of the identified drugs. The detailed structure of lipophilic, hydrophilic, and amphiphilic drugs is given in Table S4. We revealed an equal proportion of lipophilic (n = 32; 45.1%) and hydrophilic (n = 32; 45.1%) agents in PK studies in CHF patients, and a minority (n = 7; 9.9%) were amphiphilic (Table 2).

Since the structural properties of a drug govern the plasma protein binding rate, we considered PPB in this section dedicated to PCPs of drugs. Most drugs included in our analysis revealed low values of PPB (LPB; clinically non-significant values) (n = 39; 54.9%). A high rate of PPB (HPB; clinically significant values) was observed in 39.4% (n = 28). For four drugs (5.6%), relevant data on PPB could not be identified in the literature. Detailed data on the PPB of drugs included in the analysis is presented in Table 3 and Table S4.

### 3.6. Evaluation of PK Parameters in CHF Patients

As the third phase of the study, we evaluated alterations of PK parameters detected in CHF patients. Given that the route of drug administration affects the PK of a drug, subdivision of all the revealed drugs into parenterally administered and orally administered was made.

The number of parenterally administered drugs was 42: 40.5% were lipophilic (n = 17), 52.4% were hydrophilic (n = 22), and 7.1% were amphiphilic (n = 3). Demographic data derived from publications involving PK studies with parenterally administered drugs is presented in Table S5.

Analysis of PK studies, including patients receiving parenteral drugs, revealed a relatively small number of participants (mean 16.28 ± 26.16; min = 5; max = 193). The mean patient age was 59.7 ± 8.7 years (min = 18.0, max = 81.8) years. NYHA class II–IV was most frequently identified (73.9%). Left ventricle ejection fraction (LVEF) was not reported in 53.9% of cases; in 46.1%, it was ≤45%.

The number of orally administered drugs was 50: 48% were lipophilic (n = 4), 38% were hydrophilic compounds (n = 19), and 14% were amphiphilic (n = 7). Demographic characteristics from PK studies, including oral drug administration, are presented in Tables S6 and S7. The mean number of CHF patients in PK studies was 20.8 ± 32.2 (min = 3, max = 116). The mean patient age was 61.8 ± 9.9 (min = 18, max = 84) years. NYHA class II–III was the most prevalent (66.3%). LVEF data was absent in 70.7%, and in 28.3% of patients, it was ≤45%.

#### 3.6.1. Evaluation of PK Changes for Parenterally Administered Drugs

Analysis of hydrophilic drugs revealed that 62.5% exhibited LPB (a clinically non-significant rate), while 37.5% demonstrated HPB (a clinically significant rate). For further PK evaluation, we excluded studies missing the following PK parameters: clearance, volume of distribution, and half-life period. Among the total of 32 studies examining hydrophilic drugs, 2 were excluded from clearance analysis. Of the remaining 30 studies, clearance was reduced in 76.7% and increased in 23.3%. While assessing the volume of distribution, 9 of the 32 studies were excluded. Among the included 23 studies, the volume of distribution increased in 52.2% and decreased in 47.8%. The half-life period analysis excluded 8 studies, with the remaining 24 indicating a prolonged half-life in 70.8% and a reduced half-life in 29.2%.

The sum of PK changes revealed in the studies, including parenterally administered hydrophilic drugs, is shown in Table 4.

Among lipophilic drugs, LPB was predominantly observed (56.7%). Analyzing clearance changes among lipophilic agents, 4 out of 30 studies were excluded (no data on clearance). Among the remaining 26 studies, clearance decreased in 92.3% of cases and increased in only 7.7%. Assessing the volume of distribution, we excluded 11 studies (no data available). Analysis of the finally included 19 PK studies revealed a decreased volume of distribution in 52.6% and an increase in 47.4%. Half-life period evaluation resulted in the exclusion of 10 studies. Among the 20 included studies, it was prolonged in 90% and shortened in 10%.

PK changes revealed in the studies, including parenterally administered lipophilic drugs, are shown in Table 5.

Among amphiphilic drugs, LPB predominated (66.7%), clearance was reduced in 66.7%, and the volume of distribution was increased in 66.7%. In the half-life analysis, one study was excluded due to the absence of data, and 100% of the included studies demonstrated prolongation of this parameter. Detailed data is presented in Table 6.

Subsequently, we performed a correlation analysis between the PCPs of the studied drugs (hydrophilicity/lipophilicity/amphiphilicity, and PPB) and their PK (clearance, volume of distribution, and half-life).

The analysis revealed a strong positive correlation (r = 0.725; *p* = 0.00045) between the degree of PPB of lipophilic drugs and their volume of distribution. Increased PPB among lipophilic drugs correlated with an increased volume of distribution in CHF patients. A moderate positive correlation (r = 0.433; *p* = 0.0346) between PCPs (hydro-/lipophilic/amphiphilic nature) of HPB drugs and their half-life period was detected. We revealed that the lower lipophilicity of HPB drugs correlates with a reduction in the half-life period. Detailed data is presented in Table 7.

#### 3.6.2. Evaluation of PK Changes for Orally Administered Drugs

First, we studied hydrophilic drugs. Analysis of PPB rates resulted in the exclusion of eight studies. The remaining 38 studies demonstrated LPB in 71.1% of hydrophilic drugs and HPB in 29.0%. Clearance evaluation led to the exclusion of 7 out of 46 studies. Analysis disclosed decreased clearance in 76.9% and increased clearance in 23.1%. Assessing the volume of distribution, we excluded 41 studies, and only 5 were included. Analysis revealed an increased volume of distribution in 80% and a decrease in 20%. Half-life period analysis excluded 14 studies, and among the 32 remaining ones, 81.2% demonstrated prolongation, and 18.8% demonstrated reduction. A summary of PK changes of orally administered hydrophilic drugs is given in Table 8.

The analysis of lipophilic drugs predominantly revealed HPB (71.11%). Evaluating clearance, 1 study out of 45 was excluded, and the remaining 44 studies demonstrated decreased clearance in 88.6% and increased clearance in 11.4%. The volume of distribution assessment led to the exclusion of 37 studies. Among the eight included studies, decreased volume of distribution was detected in 37.5%, and an increase was detected in 62.5%. Analyzing the half-life period, 19 studies were excluded, with 26 included studies showing prolonged half-life in 80.8% and reduced half-life in 19.2%. Detailed data on PK changes revealed in the studies, including orally administered lipophilic drugs, is shown in Table 9.

Assessing the PPB of amphiphilic drugs, we excluded two studies. Among the six included studies, half of the drugs were HPB, and half were LPB. PK analysis of amphiphilic drugs demonstrated decreased clearance in 62.5% of studies and an increase in 37.5%. Assessing the volume of distribution, we excluded seven studies, with only one study included. This result demonstrated a reduction in this PK parameter. Analyzing the half-life period, three studies were excluded, and all five included studies (100%) revealed prolongation. Full data highlighting PK changes revealed in the studies, including orally administered amphiphilic drugs, is given in Table 10.

Subsequent correlation analysis between PCPs (hydrophilicity/lipophilicity/amphiphilicity and PPB) and PK parameters (clearance, volume of distribution, and half-life) revealed several significant relationships: hydrophilic drugs demonstrated a moderate positive correlation between PPB and clearance (r = 0.5044, *p* = 0.0038), where increased PPB was associated with enhanced clearance; amphiphilic compounds showed a strong inverse correlation (r = −1, *p* < 0.05) with reduced PPB resulting in increased clearance; while HPB drugs exhibited a moderate negative correlation between hydrophilicity and clearance (r = −0.3956, *p* = 0.0087). Hydrophilicity was associated with increased clearance. Detailed data is presented in Table 11.

## 4. Discussion

Our study revealed some correlations between PCPs and PK parameters of drugs in elderly patients with CHF. This study demonstrated a strong positive correlation (r = 0.725, *p* = 0.00045) between the PPB of lipophilic drugs administered parenterally and their volume of distribution. These results indicate that higher PPB is associated with increased volume of distribution for lipophilic compounds in CHF patients. This relationship can be attributed to CHF-related hypoproteinemia, which leads to elevated levels of unbound fraction with greater capacity for tissue penetration, ultimately resulting in larger distribution volumes [124]. A strong correlation between the unbound drug fraction and volume of distribution is supported by the work of K. Korzekwa et al. (2017), which examined the relationship between volume of distribution and PPB in both plasma and microsomes [125]. However, there was no link to be found for the PPB of parenteral hydrophilic and amphiphilic drugs. Considering the strong correlations revealed, we should also mention the small sample size of studies included in the final analysis. This may limit the significance of this phenomenon and require additional studies to verify its clinical value.

Furthermore, our results indicated a moderate positive correlation (r = 0.433; *p* = 0.0346) between the PCPs of HPB parenteral drugs and their half-lives. This finding suggests that decreased albumin level in CHF results in elevated unbound fraction, and lipophilic compounds exhibit prolonged half-life. This happens due to increased tissue penetration and subsequent binding with tissue proteins. This is supported by data derived from the study by H. Gunaydin et al. (2018), which concluded that reduced lipophilicity correlates with shorter half-life [126]. A high prevalence of adiposity (defined as >25% body fat in males and >35% in females) is observed in over 60% of the CHF population [127]. Patients with obesity demonstrated a direct correlation between drug lipophilicity and elimination half-life according to a study by Bruno C.D. et al. (2021) [128]. We revealed the absence of such a correlation for LPB drugs. Our study revealed a moderate negative correlation (r = −0.3956; *p* = 0.0087) between the PCPs of HPB orally administered drugs and their clearance.

Transferring our findings on other groups of drugs, we can suppose that there is a need to modify the dosing regimen in CHF patients. This may apply to such a group of antibiotics as beta-lactams, particularly cephalosporins. This class includes drugs with both HPB (e.g., cefoperazone) and LPB (e.g., ceftazidime), which may significantly affect clearance rates and consequently affect steady-state plasma drug concentrations [129].

For hydrophilic drugs, a moderate positive correlation (r = 0.504; *p* = 0.0038) was identified between PPB and clearance. In CHF patients, HPB hydrophilic drugs may demonstrate increased clearance, which stems from elevated unbound drug fractions. This happens due to hypoalbuminemia as the unbound fraction undergoes glomerular filtration [124].

A strong negative correlation (r = −1; *p* < 0.05) was observed between PPB and clearance for amphiphilic drugs, confirming that increased PPB reduces clearance in this drug category for CHF patients. This relationship may be particularly relevant for amphiphilic drugs among aminoglycosides described by Dezanet C. et al. (2020), suggesting the importance of monitoring PPB status to control the potential toxicity of these agents [130].

The limitations of our study are listed below:

- The relatively small sample size in the majority of included studies.
- The predominance of the moderate risk of bias among most of the included studies.
- Most of the included studies included data on cardiovascular drugs. Thus, extrapolation of the PK alterations to non-cardiovascular drugs remains speculative.
- Most studies were published prior to the year 2000.

Therefore, additional studies for non-cardiovascular drugs are required to improve our knowledge on PK alterations specific to CHF patients. Nevertheless, considering the pathophysiological CHF triad of reduced cardiac output, hepatic congestion, and hypoperfusion, we can propose practical recommendations. First, identification of high-risk drugs should be provided while planning treatment strategies in CHF patients (highly lipophilic, HPB). Administration of most highly lipophilic drugs should be started with a reduced dose and followed by a slow titration, allowing for sufficient time to assess both efficacy and toxicity. Some drugs, including those with a narrow therapeutic index and some antibiotics, require therapeutic drug monitoring (TDM) in CHF patients to guide therapy and avoid subtherapeutic or toxic plasma concentrations.

## 5. Conclusions

Our study revealed several consistent patterns of PK parameter changes among cardiovascular drugs with different PCPs. Based on the obtained data, the following conclusions can be drawn:

- Hypoalbuminemia associated with CHF may lead to an increased volume of distribution of lipophilic drugs.
- For lipophilic drugs used in CHF patients, potential prolongation of half-life and reduced clearance should be considered. This applies to both oral and parenteral formulations as important factors for dosing regimen modification.
- For HPB drugs used in CHF patients, reduced clearance must be considered as an important factor for dosing regimen modification.

Evaluation of correlations between drugs’ PK parameters and PCPs may provide additional insight into drug behavior in CHF patients. Some drug classes exhibit high concentration-dependent efficacy; thus, even small PK alterations may provide a pronounced effect on clinical outcomes, and antibiotics may represent an example of such drugs [131,132]. Since the data on PK changes in CHF is mainly limited to cardiovascular agents, further development of prognostic tools based on PK-PCP correlations may be helpful. Thus, additional PK studies including various pharmacological groups are required to improve our knowledge and construct proper approaches to correct dosing regimens in CHF patients.

## Figures and Tables

**Table 1:** Drug structure based on ATC classification.

ATC Drug Group	No. of Studies	%
C—Cardiovascular system	96	90.57
R—Respiratory system	5	4.72
N—Nervous system	3	2.83
B—Blood and blood forming organs	1	0.94
L—Antineoplastic and immunomodulating agents	1	0.94

**Table 2:** Drug structure according to PCPs.

PCP	N of Drugs	%	References
Hydrophilic	32	45.1	[17,18,19,20,21,22,23,24,25,26,27,28,29,30,31,32,33,34,35,36,37,38,39,40,41,42,43,44,45,46,47,48,49,50,51,52,53,54,55,56,57,58,59,60,61,62,63,64,65,66,67,68,69,70,71]
Lipophilic	32	45.1	[72,73,74,75,76,77,78,79,80,81,82,83,84,85,86,87,88,89,90,91,92,93,94,95,96,97,98,99,100,101,102,103,104,105,106,107,108,109,110,111,112,113,114,115,116]
Amphiphilic	7	9.9	[117,118,119,120,121,122,123]

**Table 3:** Drug structure according to PPB.

PPB	No. of Drugs	%	References
HPB	28	39.4	[17,26,27,28,29,38,39,40,41,42,43,44,45,59,62,66,72,73,74,75,76,77,78,81,86,88,89,90,91,92,96,97,98,103,109,110,113,114,115,116,119,123]
LPB	39	54.9	[17,18,19,20,21,22,23,24,25,32,33,34,35,36,46,47,48,49,50,51,52,53,54,55,56,57,58,71,79,80,82,83,84,85,87,93,94,95,99,104,105,106,107,108,111,112]
NA	4	5.6	[30,37,68,69,70,118]

**Table 4:** PK changes revealed in the studies, including parenterally administered hydrophilic drugs.

PK Parameter	Studies Exhibiting Decrease in PK Parameter, n (%)	Studies Exhibiting Increase in PK Parameter, n (%)	References
Clearance	23 (76.7)	7 (23.3)	[17,31,32,33,34,39,44,45,46,55,56,57,59,60,61,62,63,64,65,66,77,78,79,80,82,83,84,86,87,88,91,92,93,96,97,98,100,103,105,106,111,115,118,119,120,121,122]
Volume of distribution	11 (47.8)	12 (52.2)	[17,18,31,32,33,34,39,44,45,46,55,56,57,59,60,63,66,77,78,79,80,82,83,84,86,87,88,91,92,93,96,97,98,100,103,105,106,115,118,119,120,122]
Half-life	7 (29.2)	14 (70.8)	[17,32,33,34,39,44,45,46,55,56,57,59,60,61,62,63,66,77,79,80,81,82,83,84,86,87,88,91,92,93,96,97,98,103,105,106,115,118,119,122]

**Table 5:** PK changes revealed in the studies, including parenterally administered lipophilic drugs.

PK Parameter	Studies Exhibiting Decrease in PK Parameter, n (%)	Studies Exhibiting Increase in PK Parameter, n (%)	References
Clearance	24 (92.3)	2 (7.7)	[44,82,83,84,86,87,88,91,92,93,96,97,98,100,103,104,105,106,115,121]
Volume of distribution	10 (52.6)	9 (47.4)	[44,82,83,84,86,87,88,91,92,93,96,97,98,100,103,104,105,106,115]
Half-life	2 (10)	18 (90)	[44,81,82,83,84,86,87,88,91,92,93,96,97,98,103,104,105,106,115]

**Table 6:** PK changes revealed in the studies, including parenterally administered amphiphilic drugs.

PK Parameter	Studies Exhibiting Decrease in PK Parameter, n (%)	Studies Exhibiting Increase in PK Parameter, n (%)	References
Clearance	2 (66.7)	1 (33.3)	[119,120,122]
Volume of distribution	1 (33.3)	2 (66.7)	[119,120,122]
Half-life	NA	2 (100)	[119,122]

**Table 7:** Correlations between PCP and PK parameters for parenterally administered drugs.

	Half-Life	Volume of Distribution	Clearance
PPB of hydrophilic drugs	−0.3296 (*p* = 0.116)	−0.214 (*p* = 0.3274)	−0.0927 (*p* = 0.6262)
PPB of lipophilic drugs	0.068 (*p* = 0.776)	0.725 (*p* = 0.00045)	0.3118 (*p* = 0.121)
PPB of amphiphilic drugs	NA	NA	NA
PCP of HPB dugs	0.433 (*p* = 0.0346)	0.2175 (*p* = 0.319)	−0.055 (*p* = 0.799)
PCP of LPB drugs	0.072 (*p* = 0.749)	−0.2637 (*p* = 0.236)	−0.2002 (*p* = 0.2489)

NA-not applicable: data insufficient for correlation analysis.

**Table 8:** PK changes revealed in the studies, including orally administered hydrophilic drugs.

PK Parameter	Studies Exhibiting Decrease in PK Parameter, n (%)	Studies Exhibiting Increase in PK Parameter, n (%)	References
Clearance	30 (76.9)	9 (23.1)	[17,18,19,20,21,22,23,24,26,27,28,29,30,31,36,37,39,40,41,42,43,44,49,50,51,52,53,54,67,69,70,71,90]
Volume of distribution	1 (20)	4 (80)	[36,42,50,51]
Half-life	6 (18.8)	26 (81.2)	[17,20,21,22,24,28,30,36,37,39,40,41,43,44,47,48,49,50,67,69,70,90]

**Table 9:** PK changes revealed in the studies, including orally administered lipophilic drugs.

PK Parameter	Studies Exhibiting Decrease in PK Parameter, n (%)	Studies Exhibiting Increase in PK Parameter, n (%)	References
Clearance	39 (88.6)	5 (11.4)	[24,28,39,55,58,72,73,74,75,76,77,78,79,85,89,94,95,103,107,108,109,110,111,112,113,114,115,116,117,119]
Volume of distribution	3 (37.5)	5 (62.5)	[58,75,79,107,113]
Half-life	5 (19.2)	21 (80.8)	[24,38,39,55,58,71,76,77,78,79,81,88,89,94,95,103,107,109,111,112,113,115,119]

**Table 10:** PK changes revealed in the studies, including orally administered amphiphilic drugs.

PK Parameter	Studies Exhibiting Decrease in PK Parameter, n (%)	Studies Exhibiting Increase in PK Parameter, n (%)	References
Clearance	5 (62.5)	3 (37.5)	[29,43,44,61,101,116,117,123]
Volume of distribution	1 (100)	NA	[117]
Half-life	NA	5 (100)	[43,44,61,101,117]

NA-not applicable: no data.

**Table 11:** Correlations between PCPs and PK parameters for orally administered drugs.

	Half-Life	Volume of Distribution	Clearance
PPB of hydrophilic drugs	0.316 (*p* = 0.132)	0.25 (*p* = 0.685)	0.504 (*p* = 0.0038)
PPB of lipophilic drugs	−0.3858 (*p* = 0.052)	0.258 (*p* = 0.537)	0.2319 (*p* = 0.13)
PPB of amphiphilic drugs	NA	NA	−1 (*p* = 0)
PCP of HPB dugs	−0.2271 (*p* = 0.2645)	−0.25 (*p* = 0.685)	−0.3956 (*p* = 0.0087)
PCP of LPB drugs	0.3459 (*p* = 0.0714)	−0.258 (*p* = 0.537)	0.296 (*p* = 0.0711)

NA-not applicable: data insufficient for correlation analysis.

## Data Availability

Not applicable.

## Supplementary Materials

The following supporting information can be downloaded at: https://www.mdpi.com/article/10.3390/ijms26199495/s1.

## Abbreviations

The following abbreviations are used in this manuscript:

CHF	Chronic heart failure.
PCP	Physicochemical property.
PK	Pharmacokinetic.
PubMed	United States National Library of Medicine.
CNKI	China National Knowledge Infrastructure.
DOAJ	Directory of Open Access Journals.
PPB	Plasma protein binding.
ACE	Angiotensin-converting enzyme.
PDE	Phosphodiesterase.
NHANES	National Health and Nutrition Examination Survey.
NYHA	New York Heart Association.
ADR	Adverse drug reaction.
ATC classification	Anatomical Therapeutical Chemical classification.
NA	Not applicable.
LVEF	Left ventricle ejection fraction.
LPB	Low protein binding.
HPB	High protein binding.
PRISMA	Preferred Reporting Items for Systematic Reviews and Meta-Analyses.
