# Supplementary material for: Pharmacokinetic Alterations in Patients with Chronic Heart Failure: A Systematic Review
Source: Int J Mol Sci. 2025 Sep 28;26(19):9495. doi: 10.3390/ijms26199495 (PMC12524999; doi:10.3390/ijms26199495)
Supplement: Supplementary file 1 [file ijms-26-09495-s001.zip › ijms-3797415-supplementary.pdf]

Supplementary Materials

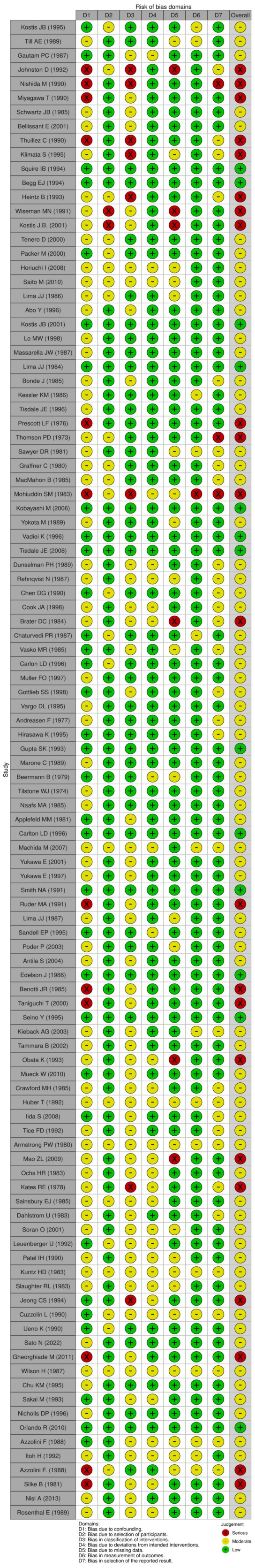

Figure S1. Quality assessment.

Table S1. All included drugs.

Drug name	N of studies	Chemical structure	References
Furosemide	9	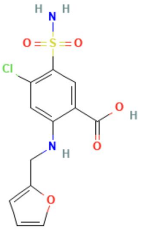	[39,40-44, 89,90,115]
Digoxin	6	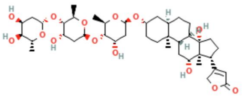	[20,48-51,88]
Theophylline	5	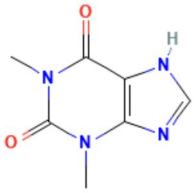	[103-107]
Milrinone	4	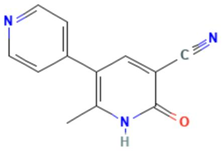	[55-57,98]
Lisinopril	3	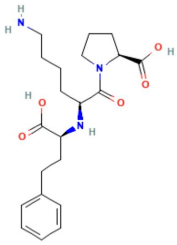	[17,18,22]
Lidocaine	3	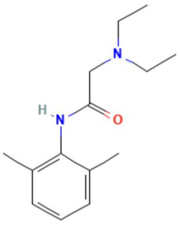	[33,82,83]
Torasemide	3	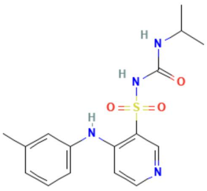	[43,46,90]
Enoximone	3	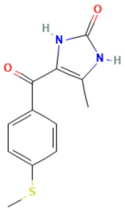	[54,93,94]

Levosimendan	3	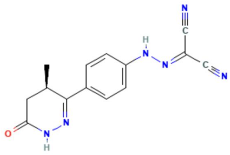	[95-97]
Ibopamine	3	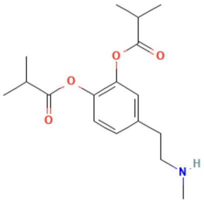	[68,69,113]
Tocainide	3	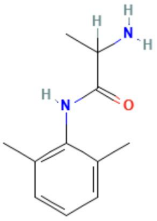	[34,35,84]
Captopril	2	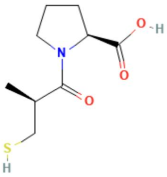	[19-20]
Enalapril	2	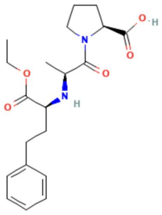	[21-22]
Enalaprilat	2	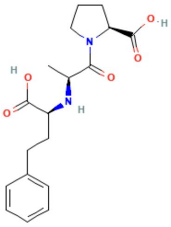	[21-22]
Perindopril	2	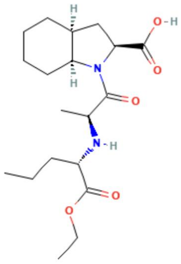	[23-24]
Perindoprilat	2	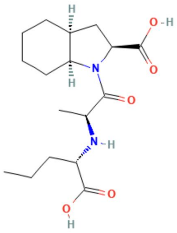	[23-24]

Quinaprilat	2	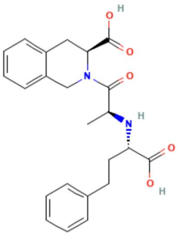	[26-27]
Disopyramide	2	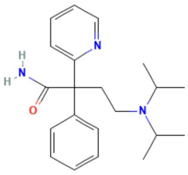	[79,118]
Procainamide	2	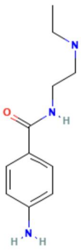	[32,81]
Felodipine	2	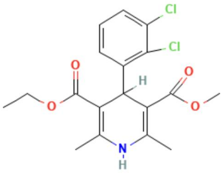	[87,88]
Bumetanide	2	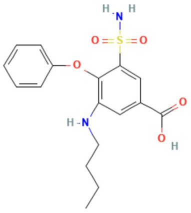	[89,115]
Flosequinan	2	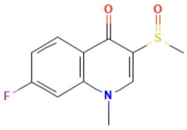	[110-112]
Nicorandil	2	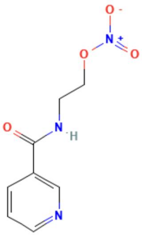	[61-120]
Prenalterol	2	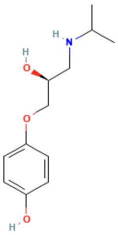	[59,60]

Carvedilol	2	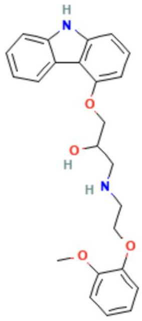	[31,72]
Fosinoprilat	1	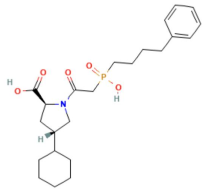	[17]
Omapatrilat	1	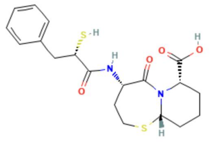	[30]
Cilazaprilat	1	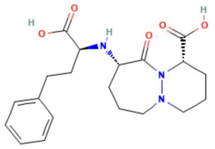	[29]
Quinapril	1	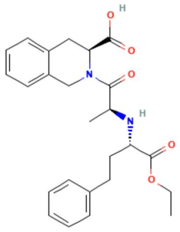	[25]
Ramipril	1	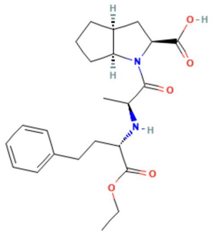	[28]
Ramiprilat	1	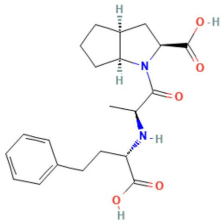	[28]
R-Carvedilol	1	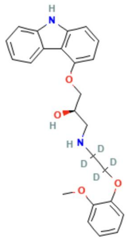	[73]

S-Carvedilol	1	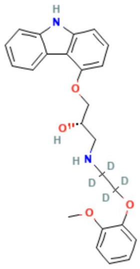	[74]
Pindolol	1	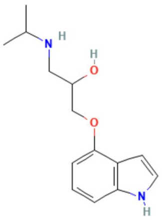	[75]
Candesartan	1	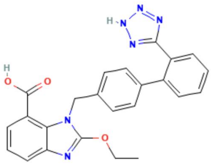	[117]
Irbesartan	1	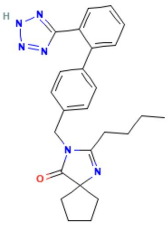	[76]
Losartan	1	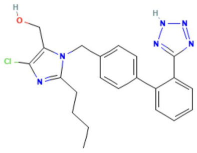	[77]
Cibenzoline	1	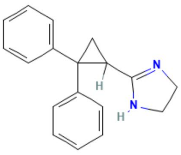	[78]
Quinidine	1	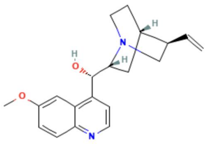	[80]
N-acetylprocainamide	1	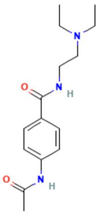	[32]
Mexiletine	1	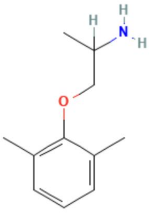	[36]

Pilsicainide	1	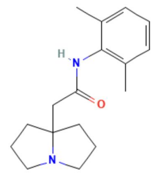	[85]
Amiodarone	1	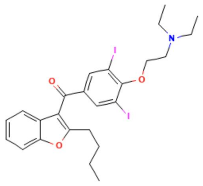	[37]
Ibutilide	1	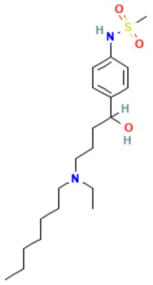	[86]
Nifedipine	1	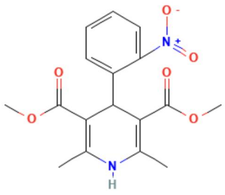	[38]
Nicardipine	1	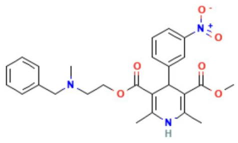	[45]
Enalkiren	1	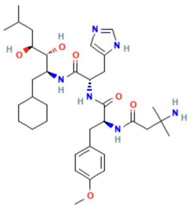	[91]
Piretanide	1	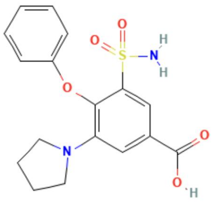	[46]
Hydrochlorothiazide	1	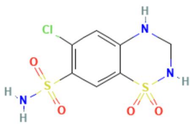	[92]
Metolazone	1	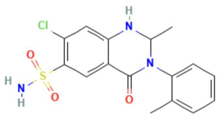	[47]

Saterinone	1	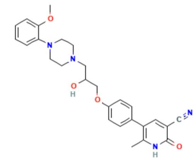	[58]
Toborinone	1	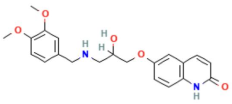	[99]
Amrinone	1	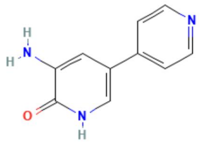	[109]
Pimobendan	1	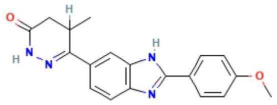	[67]
Carperitide	1	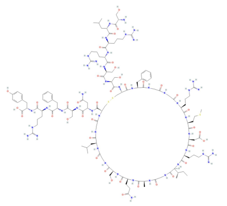	[59]
Cinaciguat	1	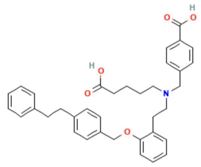	[60]
Hydralazine	1	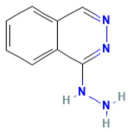	[100]
Molsidomine	1	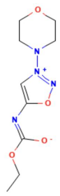	[119]
Nitroglycerin	1	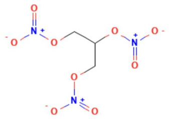	[101]
Conivaptan	1	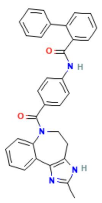	[121]
Acetaminophen	1	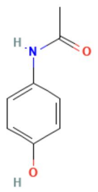	[62]

Dobutamine	1	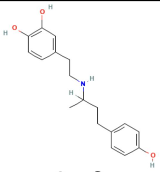	[63]
Etanercept	1	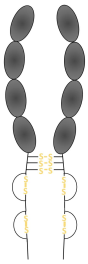	[123]
Isoproterenol	1	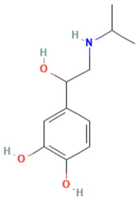	[64]
Midazolam	1	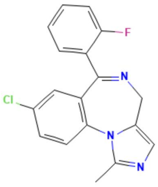	[65]
Norepinephrine	1	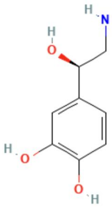	[64]
Tolvaptan	1	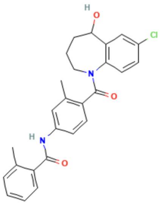	[108]
Rivaroxaban	1	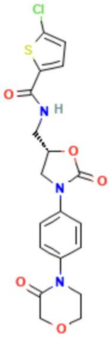	[66]
Fluvoxamine	1	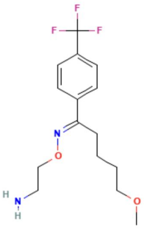	[112]

Prazosin	1	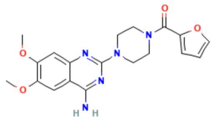	[70]
Pentoxifylline	1	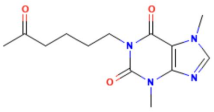	[114]

Table S2. Cardiovascular drugs.

ATC group C – Cardiovascular system	N of studies	References
C09AA ACE inhibitors, plain	15	[17-30]
C01CE Phosphodiesterase inhibitors	11	[54-58,67,93,93,98,99,109]
C03CA Sulfonamides, plain	10	[39-44,89,90,93,115]
C01CA Adrenergic and dopaminergic agents	7	[63,64,68,69,102,113]
C01BB Antiarrhythmics, class Ib	7	[33-36,82-84]
C01AA Digitalis glycosides	6	[20,48-51,88]
C01BA Antiarrhythmics, class Ia	5	[32,79-81,118]
C01DX Other vasodilators used in cardiac diseases	5	[59,60,61,119,120]
C07AG Alpha and beta blocking agents	5	[31,72-75]
C08CA Dihydropyridine derivatives	4	[38,45,87,88]
C01CX Other cardiac stimulants	3	[95-97]
C01DB Quinolone vasodilators	3	[100], [110,111]
C09CA Angiotensin II receptor blockers (ARBs), plain	3	[76,77,117]
C01BD Antiarrhythmics, class III	2	[37,86]
C03XA Vasopressin antagonists	2	[108,121]
C01BC Antiarrhythmics, class Ic	1	[85]
C01BG Other antiarrhythmics, class I and III	1	[78]
C01DA Organic nitrates	1	[101]
C02CA Alpha-adrenoreceptor antagonists	1	[70]
C03AA Thiazides, plain	1	[92]
C03BA Sulfonamides, plain	1	[47]
C04AD Purine derivatives	1	[114]
C09XA Renin-inhibitors	1	[91]

Table S3. Structure of drugs according to ATC groups.

Drugs	N of studies	References
**ATC group C – Cardiovascular system**		
Furosemide	9	[39,40-44, 89,90,115]
Digoxin	6	[20,48-51,88]
Milrinone	4	[55-57,98]
Lidocaine	3	[33,82,83]
Ibopamine	3	[68,69,113]
Enoximone	3	[54,93,94]
Levosimendan	3	[95-97]
Torasemide	3	[43,46,90]
Lisinopril	3	[17,18,22]
Tocainide	3	[34,35,84]
Disopyramide	2	[79,118]

Procainamide	2	[32,81]
Prenalterol	2	[59,60]
Flosequinan	2	[110-112]
Nicorandil	2	[61-120]
Bumetanide	2	[89,115]
Felodipine	2	[87,88]
Captopril	2	[19-20]
Enalapril	2	[21-22]
Enalaprilat	2	[21-22]
Perindopril	2	[23-24]
Perindoprilat	2	[23-24]
Quinaprilat	2	[26-27]
Carvedilol	2	[31,72]
Quinidine	1	[80]
N-acetylprocainamide	1	[32]
Mexiletine	1	[36]
Pilsicainide	1	[85]
Amiodarone	1	[37]
Ibutilide	1	[86]
Cibenzoline	1	[78]
Dobutamine	1	[63]
Isoproterenol	1	[59]
Norepinephrine	1	[64]
Saterinone	1	[58]
Toborinone	1	[99]
Amrinone	1	[109]
Pimobendan	1	[67]
Nitroglycerin	1	[101]
Hydralazine	1	[100]
Carperitide	1	[59]
Cinaciguat	1	[60]
Molsidomine	1	[118]
Prazosin	1	[70]
Hydrochlorothiazide	1	[92]
Metolazone	1	[47]
Piretanide	1	[46]
Conivaptan	1	[120]
Tolvaptan	1	[108]
Pentoxifylline	1	[114]
R-Carvedilol	1	[73]
S-Carvedilol	1	[74]
Pindolol	1	[75]
Nifedipine	1	[38]
Nicardipine	1	[45]
Fosinoprilat	1	[17]
Omapatrilat	1	[30]
Cilazaprilat	1	[29]
Quinapril	1	[26,27]
Ramipril	1	[28]
Ramiprilat	1	[28]
Candesartan	1	[117]
Irbesartan	1	[76]
Losartan	1	[77]

Enalkiren	1	[91]
**ATC group R – Respiratory system**		
Theophylline	5	[103-107]
**ATC group N – Nervous system**		
Acetaminophen	1	[62]
Midazolam	1	[65]
Fluvoxamine	1	[112]
**ATC group B – Blood and blood forming organs**		
Rivaroxaban	1	[66]
**ATC group L – Antineoplastic and immunomodulating agents**		
Etanercept	1	[123]

Table S4. Drugs structure based on hydrophilicity, lipophilicity, amphiphilicity.

PK property	Drug name	References
Hydrophilic	Acetaminophen<br>Amrinone<br>Captopril<br>Carperitide<br>Cilazaprilat<br>Conivaptan<br>Digoxin<br>Enalapril<br>Enalaprilat<br>Enalkiren<br>Etanercept<br>Fosinoprilat<br>Furosemide<br>Hydrochlorothiazide<br>Ibopamine<br>Isoproterenol<br>Lisinopril<br>Metolazone<br>Milrinone<br>Molsidomine<br>N-acetylprocainamide<br>Norepinephrine<br>Omapatrilat<br>Pentoxifylline<br>Perindoprilat<br>Pilsicainide<br>Procainamide<br>Quinaprilat<br>Ramiprilat<br>Toborinone<br>Tocainide<br>Tolvaptan	[17-71]
Lipophilic	Amiodarone<br>Bumetanide	[72-116]

	Candesartan<br>Carvedilol<br>Cinaciguat<br>Disopyramide<br>Dobutamine<br>Enoximone<br>Felodipine<br>Flosequinan<br>Fluvoxamine<br>Ibutilide<br>Irbesartan<br>Levosimendan<br>Lidocaine<br>Losartan<br>Mexiletine<br>Midazolam<br>Nicardipine<br>Nifedipine<br>Nitroglycerin<br>Perindopril<br>Pimobendan<br>Piretanide<br>Prazosin<br>Quinapril<br>Quinidine<br>R-Carvedilol<br>Rivaroxaban<br>S-Carvedilol<br>Saterinone<br>Theophylline	
Amphiphilic	Cibenzoline<br>Hydralazine<br>Nicorandil<br>Pindolol<br>Prenalterol<br>Ramipril<br>Torasemide	[117-123]

Table S5. Demographic data for parenterally administered drugs.

Drug	CHF/control	Mean age	NYHA class	LVEF	References
Fosinoprilat	10/10	77.8+9.5	II/III	32	[16]
Lisinopril	12/0	57+16.5	III/IV	NA	[17]
Omapatrilat	19/17	55+16	II/III	23	[29]
Irbesartan	10/10	>18	II/III	26	[117]
Losartan	11/0	47+13	II/III/IV	27	[76]
Cibenzoline	6/5	58+11	II/III	28	[77]
Disopyramide	7/11	>18	NA	NA	[118]
Disopyramide	6/11	35.8+7.36	NA	NA	[78]

Disopyramide	11/5	35.8+7.36	II/III/IV	25	[78]
Procainamide	15/10	57+12	I/II/III	30	[81]
Procainamide	9/7	61.4+12.7	II/III	22	[32]
N-acetylprocainamide	9/7	61.4+12.7	II/III	22	[32]
Quinidine	7/9	>18	NA	NA	[32]
Lidocaine	7/6	57.7+10	NA	NA	[33]
Lidocaine	8/10	64+20	NA	NA	[82]
Lidocaine	3/10	23+4.0	NA	NA	[83]
Lidocaine	7/10	23+4.0	NA	NA	[83]
Tocainide	14/0	64,8+10	NA	NA	[84]
Amiodarone	6/11	50±10	II/III	35	[85]
Amiodarone	6/11	50±10	II/III	25	[85]
Ibutilide	6/10	53±17	II/III	30	[86]
Felodipine	11/0	62±7	III	<40	[87]
Nicardipine	5/0	>18	III/IV	NA	[44]
Enalkiren	12/0	73±12	NA	21	[91]
Bumetanide	6/4	51.8±14.9	III/IV	20	[89]
Furosemide	18/8	50.5±11.75	NA	NA	[43]
Furosemide	16/8	>18	NA	32	[115]
Furosemide	16/0	59,8±11.6	II/III	<40	[90]
Piretanide	6/0	71.7±10	NA	NA	[46]
Torasemide	16/0	50.5±11.75	II/III	<40	[43]
Enoximone	21/0	57.44±13.3	II/III/IV	<45	[54]
Levosimendan	8/8	>18	II	30	[95]
Levosimendan	193/0	>18	III/IV	NA	[95]
Levosimendan	29/0	>18	III/IV	31	[96]
Levosimendan	24/0	>18	III/IV	NA	[97]
Milrinone	26/0	62±9.5	III/IV	NA	[98]
Milrinone	13/0	56±13.2	III/IV	8-27	[55]
Milrinone	6/0	65.6±11.2	IV	NA	[56]
Saterinone	12/0	56,6±13.2	III	NA	[57]
Toborinone	10/0	56.8±13.3	II/III	26	[58]
Toborinone	11/0	56.8±13.3	II/III/IV	26	[58]
Toborinone	5/0	56.8±13.3	II/III	22	[58]
Toborinone	6/0	56.8±13.3	III/IV	30	[58]
Carperitide	10/0	>18	III/IV	NA	[63]
Cinaciguat	56/0	66±11.5	III/IV	NA	[59]
Hydralazine	10/0	59.5±5.25	III	NA	[60]
Molsidomine	10/0	>18	NA	NA	[100]
Nicorandil	46/6	65.1±11.5	II/III/IV	NA	[119]
Nitroglycerin	16/0	55.5±9.2	III/IV	NA	[120]
Conivaptan	5/11	73,1±14.3	NA	NA	[101]
Acetaminoph	12/12	48±3	II/III/IV	NA	[121]

en					
Dobutamine	7/0	54.1+12.8	IV	NA	[63]
Etanercept	11/0	52,5+10.75	II/III/IV	NA	[122]
Isoproterenol	8/9	56.1+10.8	III/IV	20	[123]
Midazolam	6/6	49.5+5.75	II/III	19	[64]
Norepinephrine	8/9	56.1+10.8	III/IV	20	[112]
Prenalterol	5/0	57+3.3	II/III	20	[63]
Theophylline	50/20	46+13	III/IV	NA	[65]
Theophylline	18/39	75+8	II/III/IV	NA	[103]
Theophylline	15/34	75+8	II/III/IV	NA	[103]
Theophylline	10/16	75+8	II/III/IV	NA	[103]
Theophylline	6/12	75+8	II/III/IV	NA	[103]
Theophylline	8/0	67,9+5.9	IV	NA	[104]
Theophylline	8/0	67,9+5.9	I/II	NA	[104]
Tolvaptan	74/0	52,5+16.25	I/II/III/IV	NA	[107]

Table S6. Demographic data for orally administered drugs.

Drug	CHF/control	Mean age	NYHA class	LVEF	References
Captopril	7/0	60+11.2	II	NA	[19]
Captopril	5/0	60+11.2	III/IV	NA	[19]
Captopril	12/0	53,8+5.0	I/II	NA	[20]
Cilazaprilat	11/0	49+15.5	II/III	NA	[29]
Cilazaprilat	10/0	57+4.5	II/III	NA	[114]
Enalapril	8/4	53,4+8.6	III/IV	NA	[21]
Enalapril	7/0	53,4+8.6	III/IV	NA	[21]
Enalaprilat	7/0	53,4+8.6	III/IV	NA	[21]
Enalaprilat	8/4	53,4+8.6	III/IV	NA	[21]
Fosinoprilat	10/10	65+15.5	II/III	32	[15]
Lisinopril	12/0	57+16.5	III/IV	NA	[17]
Lisinopril	5/6	>18	NA	NA	[18]
Lisinopril	6/0	>18	III/IV	NA	[22]
Omapatrilat	19/17	49+15.5	II/III	23	[29]
Perindopril	10/6	64,4+8.3	III/IV	NA	[23]
Perindoprilat	10/6	64,4+8.3	III/IV	NA	[23]
Quinapril	11/0	65+7	II/III/IV	NA	[26]
Quinaprilat	12/0	72+8.5	II/III	NA	[25]
Quinaprilat	12/0	65+7	II/III	35-50 (n=6); <35 (n=6)	[26]
Quinaprilat	11/0	>18	II/III/IV	NA	[24]
Ramipril	12/0	>18	II/III	NA	[27]
Ramiprilat	12/0	>18	II/III	NA	[27]
Carvedilol	10/0	55+6.25	III	NA	[30]
Carvedilol	10/0	55+6.25	IV	NA	[30]
Carvedilol	173/0	51,5+16.75	NA	26	[31]
Carvedilol	24/0	70,5+11.3	NA	NA	[72]
Carvedilol	58/0	65+14.0	I-IV	29,4	[73]
R-Carvedilol	10/0	55+6.25	III	NA	[30]
R-Carvedilol	10/0	55+6.25	IV	NA	[30]

R-Carvedilol	173/0	51,5±16.75	NA	26	[31]
R-Carvedilol	24/0	70,5±11.3	NA	NA	[72]
R-Carvedilol	58/0	65±14.0	I-IV	NA	[73]
S-Carvedilol	10/0	55±6.25	III	NA	[30]
S-Carvedilol	10/0	55±6.25	IV	NA	[30]
S-Carvedilol	173/0	51,5±16.75	NA	26	[31]
S-Carvedilol	24/0	70,5±11.3	NA	NA	[72]
S-Carvedilol	58/0	65±14.0	I-IV	NA	[73]
Pindolol	8/0	47±9.25	II/III	<45	[74]
Candesartan	5/0	>18	II/III	NA	[75]
Irbesartan	10/10	>18	II/III	26	[117]
Losartan	11/0	47±13	II/III/IV	27	[76]
Cibenzoline	6/5	58±11	II/III	28	[77]
Disopyramide	11/5	35.8±7.36	II/III/IV	25	[78]
Disopyramide	11/5	35.8±7.36	II/III/IV	25	[78]
Quinidine	7/9	>18	NA	NA	[79]
Mexiletine	116/374	61.9±15.1	I/II	NA	[35]
Mexiletine	96/374	58.7±15.2	III/IV	NA	[35]
Pilsicainide	3/14	>18	II/III	29	[36]
Tocainide	7/7	>18	II-IV	NA	[34]
Digoxin	10/9	77±11	NA	NA	[47]
Digoxin	8/0	62,1±9.25	NA	NA	[48]
Digoxin	8/0	62,1±9.25	NA	NA	[48]
Digoxin	30/0	59±9.8	III/IV	<30	[49]
Digoxin	14/92	60,5±12.4	NA	NA	[51]
Digoxin	77/308	60,47±13.45	NA	NA	[52]
Digoxin	9/0	>18	II/III	NA	[50]
Felodipine	11/0	62±7.0	III	<40	[86]
Felodipine	11/0	64.6±2.25	II/III	NA	[87]
Nifedipine	12/5	>18	III/IV	NA	[88]
Bumetanide	6/4	60±12.0	III/IV	20	[38]
Bumetanide	20/0	53.5±10.25	I-IV	NA	[89]
Furosemide	20/0	53.5±10.25	I-IV	NA	[89]
Furosemide	10/0	>18	NA	NA	[115]
Furosemide	11/0	62.5±9.9	III	NA	[39]
Furosemide	11/0	62.5±9.9	III	NA	[39]
Furosemide	23/0	>18	III/IV	NA	[40]
Furosemide	19/0	65±6.5	II/III	NA	[41]
Furosemide	18/0	58±11.0	III/IV	<40	[42]
Furosemide	16/0	59,75±11.6	II/III	<40	[90]
Torasemide	19/0	58±11.0	III/IV	<40	[42]
Torasemide	16/0	59,75±11.6	II/III	<40	[90]
Rivaroxaban	6/0	52.5±13.16	III	22	[108]
Rivaroxaban	12/0	58.9±15.86	III/IV	21	[108]
Amrinone	15/0	>18	II/III/IV	NA	[66]
Enoximone	10/0	57,4±13.25	II/III/IV	NA	[54]
Enoximone	7/2	51,5±12.5	III/IV	<45	[93]
Milrinone	26/0	>18	III/IV	NA	[97]
Milrinone	22/13	51.5±13.0	NA	NA	[56]
Pimobendan	8/0	55±9.0	III/IV	18	[109]

Hydrochlorothiazide	7/0	71,7±10.0	NA	NA	[46]
Metolazone	3/3	>18	NA	NA	[92]
Flosequinan	8/0	84±5.5	II/III	NA	[67]
Flosequinan	18/0	72,9±10.0	II/III/IV	NA	[110]
Hydralazine	10/0	59.5±5.25	III	NA	[60]
Molsidomine	9/0	>18	NA	NA	[100]
Nicorandil	25/0	51±12.25	II-IV	NA	[61]
Fluvoxamine	10/10	79±6.0	III/IV	31	[111]
Ibopamine	10/0	>18	II/III/IV	NA	[112]
Ibopamine	20/0	62,7±9.0	II	NA	[113]
Ibopamine	9/8	>18	IV	23	[68]
Midazolam	6/6	46±5.75	II/III	NA	[64]
Prazosin	8/0	55,5±5.75	III/IV	NA	[69]
Prenalterol	12/0	77,8±10.8	III/IV	NA	[102]
Theophylline	11/15	77±10.5	II/III	NA	[105]
Theophylline	16/16	>18	>II	NA	[106]
Pentoxifylline	20/0	65±10	III/IV	NA	[70]

Table S7. PK parameters of drugs revealed in the study.

Drug Name	Administration route	PPB	CL	Vd	T 1/2	References
Fosinoprilat	Parenteral	High	↓	↓	↓	[16]
Lisinopril	Parenteral	Low	↓	NA	NA	[17]
Omapatrilat	Parenteral	Low	↑	↓	NA	[29]
Irbesartan	Parenteral	High	↓	↓	↑	[117]
Losartan	Parenteral	High	↓	NA	↑	[76]
Cibenzoline	Parenteral	NA	↓	↓	NA	[77]
Disopyramide	Parenteral	Low	↓	↑	↑	[118]
Disopyramide	Parenteral	Low	↓	↑	↑	[78]
Disopyramide	Parenteral	Low	↓	↑	↑	[78]
Procainamide	Parenteral	Low	↓	↓	↑	[81]
Procainamide	Parenteral	Low	↓	↓	↑	[32]
N-acetylprocainamide	Parenteral	Low	NA	NA	↓	[32]
Quinidine	Parenteral	High	NA	NA	↑	[32]
Lidocaine	Parenteral	Low	NA	NA	↑	[33]
Lidocaine	Parenteral	Low	NA	NA	↑	[82]
Lidocaine	Parenteral	Low	NA	NA	↑	[83]
Lidocaine	Parenteral	Low	NA	NA	↑	[83]
Tocainide	Parenteral	Low	↑	↑	↑	[84]
Amiodarone	Parenteral	High	↓	↑	↑	[85]
Amiodarone	Parenteral	High	↓	↑	↑	[85]
Ibutilide	Parenteral	Low	↓	↓	↑	[86]
Felodipine	Parenteral	High	↓	NA	↑	[87]
Nicardipine	Parenteral	High	↑	↑	↑	[44]
Enalkiren	Parenteral	Low	↓	↑	↑	[91]
Bumetanide	Parenteral	High	↓	NA	↑	[89]
Furosemide	Parenteral	High	NA	NA	↑	[43]
Furosemide	Parenteral	High	NA	NA	↑	[115]
Furosemide	Parenteral	High	NA	NA	↑	[90]
Piretanide	Parenteral	High	↓	↓	↑	[46]
Torasemide	Parenteral	High	↓	NA	↑	[43]

Enoximone	Parenteral	Low	↓	↓	↓	[54]
Levosimendan	Parenteral	High	↓	↓	↑	[95]
Levosimendan	Parenteral	High	↓	↓	↑	[95]
Levosimendan	Parenteral	High	↓	↓	↑	[96]
Levosimendan	Parenteral	High	↓	↓	↑	[97]
Milrinone	Parenteral	Low	↓	↑	↑	[98]
Milrinone	Parenteral	Low	↓	↑	↑	[55]
Milrinone	Parenteral	Low	↓	↑	↑	[56]
Saterinone	Parenteral	Low	NA	NA	NA	[57]
Toborinone	Parenteral	High	↓	↑	↓	[58]
Toborinone	Parenteral	High	↓	↑	↓	[58]
Toborinone	Parenteral	High	↓	↑	↓	[58]
Toborinone	Parenteral	High	↓	↑	↓	[58]
Carperitide	Parenteral	Low	↑	↑	↑	[63]
Cinaciguat	Parenteral	Low	↓	↓	NA	[59]
Hydralazine	Parenteral	High	↓	↓	↑	[60]
Molsidomine	Parenteral	Low	↓	NA	↑	[100]
Nicorandil	Parenteral	Low	↑	↑	NA	[118]
Nitroglycerin	Parenteral	Low	↓	NA	NA	[119]
Conivaptan	Parenteral	High	↑	NA	↑	[101]
Acetaminophen	Parenteral	Low	↓	↓	↑	[120]
Dobutamine	Parenteral	Low	NA	NA	NA	[63]
Etanercept	Parenteral	Low	↑	NA	NA	[122]
Isoproterenol	Parenteral	Low	↓	NA	NA	[123]
Midazolam	Parenteral	High	↓	↑	↑	[64]
Norepinephrine	Parenteral	Low	↓	NA	NA	[112]
Prenalterol	Parenteral	Low	↓	↑	↑	[63]
Theophylline	Parenteral	Low	↓	NA	↑	[65]
Theophylline	Parenteral	Low	↓	NA	↑	[103]
Theophylline	Parenteral	Low	↓	NA	↑	[103]
Theophylline	Parenteral	Low	↓	NA	↑	[103]
Theophylline	Parenteral	Low	↓	NA	↑	[103]
Theophylline	Parenteral	Low	↓	NA	↑	[104]
Theophylline	Parenteral	Low	↓	NA	↑	[104]
Tolvaptan	Parenteral	High	NA	NA	↓	[107]
Captopril	Oral	Low	↓	↓	↓	[19]
Captopril	Oral	Low	↓	NA	NA	[19]
Captopril	Oral	Low	↑	↓	NA	[20]
Cilazaprilat	Oral	NA	↓	NA	↑	[29]
Cilazaprilat	Oral	NA	↓	NA	↑	[114]
Enalapril	Oral	Low	↓	NA	↑	[21]
Enalapril	Oral	Low	↑	NA	NA	[21]
Enalaprilat	Oral	Low	↓	NA	↑	[21]
Enalaprilat	Oral	Low	↓	NA	↑	[21]
Fosinoprilat	Oral	High	↓	↓	↓	[15]
Lisinopril	Oral	Low	↓	NA	NA	[17]
Lisinopril	Oral	Low	↓	NA	NA	[18]
Lisinopril	Oral	Low	↓	NA	NA	[22]
Omapatrilat	Oral	Low	↑	↓	NA	[29]
Perindopril	Oral	Low	↓	NA	↑	[23]
Perindoprilat	Oral	Low	↓	NA	↓	[23]
Quinapril	Oral	High	↓	NA	NA	[26]
Quinaprilat	Oral	High	↑	NA	NA	[25]

Quinaprilat	Oral	High	↑	NA	↑	[26]
Quinaprilat	Oral	High	↑	NA	NA	[24]
Ramipril	Oral	Low	↑	NA	NA	[27]
Ramiprilat	Oral	Low	↓	NA	NA	[27]
Carvedilol	Oral	High	↓	NA	NA	[30]
Carvedilol	Oral	High	↓	NA	NA	[30]
Carvedilol	Oral	High	↓	NA	NA	[31]
Carvedilol	Oral	High	↓	NA	NA	[72]
Carvedilol	Oral	High	↓	NA	NA	[73]
R-Carvedilol	Oral	High	↓	NA	NA	[30]
R-Carvedilol	Oral	High	↓	NA	NA	[30]
R-Carvedilol	Oral	High	↓	NA	NA	[31]
R-Carvedilol	Oral	High	↓	NA	NA	[72]
R-Carvedilol	Oral	High	↓	NA	NA	[73]
S-Carvedilol	Oral	High	↓	NA	NA	[30]
S-Carvedilol	Oral	High	↓	NA	NA	[30]
S-Carvedilol	Oral	High	↓	NA	NA	[31]
S-Carvedilol	Oral	High	↓	NA	NA	[72]
S-Carvedilol	Oral	High	↓	NA	NA	[73]
Pindolol	Oral	Low	↑	NA	↑	[74]
Candesartan	Oral	High	↓	NA	↑	[75]
Irbesartan	Oral	High	↓	↓	↑	[117]
Losartan	Oral	High	↓	NA	↑	[76]
Cibenzoline	Oral	NA	↓	↓	NA	[77]
Disopyramide	Oral	Low	↓	↑	↑	[78]
Disopyramide	Oral	Low	↓	↑	↑	[78]
Quinidine	Oral	High	NA	NA	↑	[79]
Mexiletine	Oral	Low	↓	NA	NA	[35]
Mexiletine	Oral	Low	↓	NA	NA	[35]
Pilsicainide	Oral	NA	↓	NA	↑	[36]
Tocainide	Oral	Low	↑	↑	↑	[34]
Digoxin	Oral	Low	↓	NA	NA	[47]
Digoxin	Oral	Low	↓	NA	NA	[48]
Digoxin	Oral	Low	↓	NA	NA	[48]
Digoxin	Oral	Low	↓	NA	NA	[49]
Digoxin	Oral	Low	↓	NA	NA	[51]
Digoxin	Oral	Low	↓	NA	NA	[52]
Digoxin	Oral	Low	↓	NA	NA	[50]
Felodipine	Oral	High	↓	NA	↑	[86]
Felodipine	Oral	High	↓	NA	↑	[87]
Nifedipine	Oral	High	↑	NA	↓	[88]
Bumetanide	Oral	High	↓	NA	↑	[38]
Bumetanide	Oral	High	↓	NA	↑	[89]
Furosemide	Oral	High	NA	NA	↑	[89]
Furosemide	Oral	High	NA	NA	↑	[115]
Furosemide	Oral	High	NA	NA	↑	[39]
Furosemide	Oral	High	NA	NA	↑	[39]
Furosemide	Oral	High	NA	NA	↑	[40]
Furosemide	Oral	High	NA	NA	↑	[41]
Furosemide	Oral	High	NA	NA	↑	[42]
Furosemide	Oral	High	NA	NA	↑	[90]
Torasemide	Oral	High	↓	NA	↑	[42]
Torasemide	Oral	High	↓	NA	↑	[90]

Rivaroxaban	Oral	High	↓	NA	↓	[108]
Rivaroxaban	Oral	High	↓	NA	↓	[108]
Amrinone	Oral	Low	↓	NA	↑	[66]
Enoximone	Oral	Low	↓	↓	↓	[54]
Enoximone	Oral	Low	↓	↓	↓	[93]
Milrinone	Oral	Low	↓	↑	↑	[97]
Milrinone	Oral	Low	↓	↑	↑	[56]
Pimobendan	Oral	High	↑	NA	↑	[109]
Hydrochlorothiazide	Oral	Low	NA	NA	↑	[46]
Metolazone	Oral	Low	NA	NA	↑	[92]
Flosequinan	Oral	Low	↓	NA	↑	[67]
Flosequinan	Oral	Low	↓	NA	↑	[110]
Hydralazine	Oral	High	↓	↓	↑	[60]
Molsidomine	Oral	Low	↓	NA	↑	[100]
Nicorandil	Oral	Low	↑	↑	NA	[61]
Fluvoxamine	Oral	High	↓	↓	↑	[111]
Ibopamine	Oral	NA	↓	NA	↓	[112]
Ibopamine	Oral	NA	↓	NA	↓	[113]
Ibopamine	Oral	NA	↓	NA	↓	[68]
Midazolam	Oral	High	↓	↑	↑	[64]
Prazosin	Oral	High	↓	NA	↑	[69]
Prenalterol	Oral	Low	↓	↑	↑	[102]
Theophylline	Oral	Low	↓	NA	↑	[105]
Theophylline	Oral	Low	↓	NA	↑	[106]
Pentoxifylline	Oral	Low	↓	NA	NA	[70]
